# A Comparison of Case Fatality Risk of COVID-19 between Singapore and Japan

**DOI:** 10.3390/jcm9103326

**Published:** 2020-10-16

**Authors:** Taishi Kayano, Hiroshi Nishiura

**Affiliations:** 1 School of Public Health, Kyoto University, Yoshida-Konoe-cho, Sakyo-ku, Kyoto 606-8501, Japan; kayano.taishi.2w@kyoto-u.ac.jp; 2 Core Research for Evolutional Science and Technology (CREST), Japan Science and Technology Agency, Honcho 4-1-8, Kawaguchi, Saitama 332-0012, Japan

**Keywords:** mortality, emerging infectious disease, statistical estimation, virulence, severity

## Abstract

The crude case fatality risk (CFR) for coronavirus disease (COVID-19) in Singapore is remarkably small. We aimed to estimate the unbiased CFR by age for Singapore and Japan and compare these estimates by calculating the standardized mortality ratio (SMR). Age-specific CFRs for COVID-19 were estimated in real time, adjusting for the delay from illness onset to death. The SMR in Japan was estimated by using the age distribution of the Singapore population. Among cases aged 60–69 years and 70–79 years, the age-specific CFRs in Singapore were estimated as 1.84% (95% confidence interval: 0.46–4.72%) and 5.57% (1.41–13.97%), respectively, and those in Japan as 5.52% (4.55–6.62%) and 15.49% (13.81–17.27%), respectively. The SMR of COVID-19 in Japan, when compared with Singapore as the baseline, was estimated to be 1.46 (1.09–2.96). The overall CFR for Singapore is lower than that for Japan. It is possible that the circulating variant of severe acute respiratory syndrome coronavirus 2 (SARS-CoV-2) in Singapore causes a milder clinical course of COVID-19 infection compared with other strains. If infection with a low-virulence SARS-CoV-2 variant provides protection against infection by high-virulence strains, the existence of such a strain is encouraging news for the many countries struggling to suppress this virus.

## 1. Introduction

**Figure 1:**
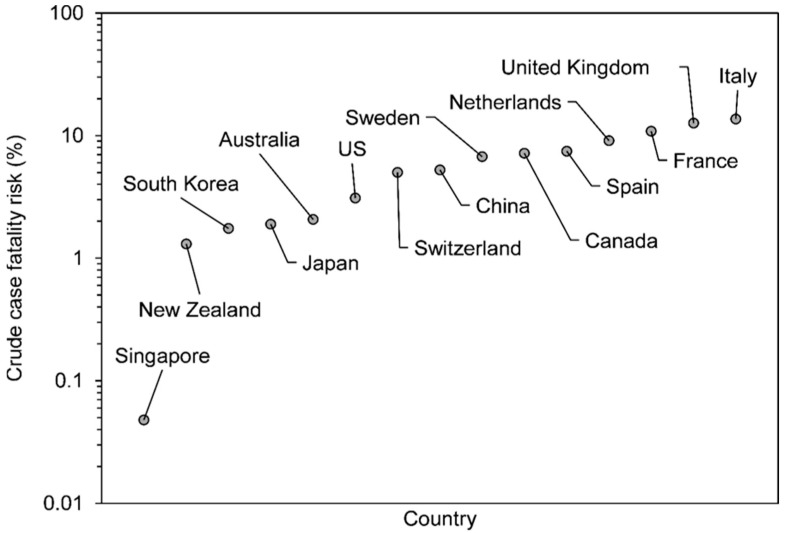
Crude case fatality risk of 2019 novel coronavirus disease (COVID-19) by country. The vertical axis represents the crude CFR value of COVID-19. Countries are ordered from the lowest to highest according to the CFR estimate, mostly sampled from high-income countries with surveillance systems to count the number of confirmed COVID-19 cases.

The global pandemic of the 2019 novel coronavirus disease (COVID-19) is currently ongoing, partially fueled by a substantial number of imported cases from other countries via international travel. The clinical impact of COVID-19 appears to vary among countries, and sound biological explanations for this observation are still lacking. The case fatality risk (CFR) plays a central role in epidemiologically measuring the risk of death. Figure 1 illustrates the variation among crude CFR estimates for COVID-19, which are calculated as the cumulative number of deaths from COVID-19 divided by the cumulative number of confirmed cases of COVID-19 on a particular date, by country [1]. Regarding COVID-19 CFR estimates for high-income countries, the crude CFR of Singapore is remarkably low compared with those of other countries.

There are several potential reasons for the small COVID-19 CFR estimate for Singapore. First, the age distribution of the Singapore population may have affected this group’s COVID-19 outcomes. It is widely recognized that older persons are more vulnerable to COVID-19 when compared with younger individuals [2]. Thus, the number of deaths in a population should be lower in populations with a higher proportion of young people. Second, Singapore may have a higher proportion of people with COVID-19 who were successfully traced and diagnosed as confirmed cases. Because of ascertainment bias, estimations of confirmed CFR for COVID-19 can be sensitive to the epidemiological surveillance capacity [3]. In Singapore, where contact tracing has been practiced very extensively, the implemented public health measures may have had an impact on the COVID-19 CFR estimate. Third, people in Singapore may possess pre-existing immunity against COVID-19. However, the idea of regional variations in pre-existing immunity against COVID-19 remains controversial, and this possibility has not yet been validated [4,5].

The present study aimed to estimate the unbiased age-dependent CFRs for Singapore and Japan and calculate the standardized mortality ratio (SMR). Japan was selected as a comparison group because the cumulative number of deaths has been maintained as being low compared with Western countries while the CFR has been estimated to be comparable to those countries. The results were used to assess the likelihood of the above-mentioned hypotheses.

## 2. Materials and Methods

### 2.1. Epidemiological Data

In Singapore, the incidence and death data of confirmed COVID-19 cases were obtained from the website of the Ministry of Health (MOH) in Singapore [6]. As there have been no further updates of COVID-19 case details (i.e., sex and ages) since 20 April 2020, our dataset includes cases up to 19 April 2020. To quantify the age distribution of the COVID-19 cases, the cases were divided into nine different age groups: 0–19, 20–29, 30–39, 40–49, 50–59, 60–69, 70–79, 80–89, and ≥90 years old. For the COVID-19 cases in Japan, we analyzed the incidence and death data of confirmed cases reported to the Ministry of Health, Labour and Welfare (MHLW). The date of illness onset and the date of death were computed. All included COVID-19 cases were divided into the same age groups as those used for the Singapore cases. We computed the confirmed COVID-19 cases and deaths of patients with an illness onset between 1 January 2020 and 31 May 2020, which is regarded as the course of the first wave of the COVID-19 epidemic in Japan. 

### 2.2. Statistical Estimation

The standard estimation of a CFR during the course of an epidemic is right-censored owing to the delay from the illness onset to death [7]. To obtain an unbiased estimate of the CFR, the incidence, p, which is the denominator of the CFR calculation, was adjusted by the probability mass function of the delay from illness onset to death, h(s), which was assumed to follow a gamma (Japan) or lognormal (Singapore) distribution. Because of the scarcity of data on illness onset for cases ending in death in Singapore, the dates of COVID-19 confirmation were used to impute the dates of illness onset. The following likelihood equation was employed:

(1) $$L\left(p_{ag};c_{a,t},\theta \right)=\underset{t}{\prod }\left(\begin{array}{c} \sum_{t=1}^{t}c_{a,t}\\ D_{a} \end{array}\right){\left(p_{a,g}\frac{\sum_{t=2}^{t}\sum_{s=1}^{t-1}c_{a,t-s}h_{s}}{\sum_{t=1}^{t}c_{a,t}}\right)}^{D_{a}}{\left(1-p_{a,g}\frac{\sum_{t=2}^{t}\sum_{s=1}^{t-1}c_{a,t-s}h_{s}}{\sum_{t=1}^{t}c_{a,t}}\right)}^{\sum_{t=1}^{t}c_{a,t}-D_{a}}\;$$

where $p_{a,g}$ is the unbiased CFR of the age group a and g={s,j} stands for country, with s and j representing Singapore and Japan, respectively. $c_{a,t}$ represents the cases of the age group a who showed illness onset (Japan) or were confirmed (Singapore) on calendar day t, and $D_{a}$ is the total number of observed deaths of the age group a. Because there were many gaps in the data on illness onset in Japan, we back-projected the illness onset as the COVID-19 confirmation day minus the estimated delay from illness onset to COVID-19 confirmation, which was sampled from the gamma distribution with a mean of 7.6 days to avoid underestimating the cases. By minimizing the negative logarithm of (1), the CFR was estimated. The 95% confidence interval (CI) of each CFR was computed by using the profile likelihood.

Subsequently, we calculated the SMR, which is commonly used for comparisons between different healthcare settings and/or demographics [8]. To compare the COVID-19 CFR of Japan to that of Singapore, we calculated the SMR as:

(2) $$SMR=\frac{d_{j}}{\sum_{a=1}^{n_{a}}p_{a,s}C_{a,j}}$$

where $d_{j}$ is the total number of observed deaths from COVID-19 in Japan, $p_{as}$ is the unbiased CFR of the age group a in Singapore estimated by Equation (1), and $C_{a,j}$ represents the total number of COVID-19 cases of the age group a in Japan, i.e., the denominator is regarded as the expected number of cases in Japan adjusted by the age distribution of confirmed cases in Singapore. The 95% CI of the SMR was computed by using a parametric bootstrap method. A total of 1000 sets of resampling were performed to calculate the 2.5th and 97.5th percentile values of the resampled distribution. 

### 2.3. Ethical Considerations

All datasets used in this study are publicly available, and the personal data of cases were deidentified before we obtained the datasets. Accordingly, the present study did not require ethical approval.

## 3. Results

**Figure 2:**
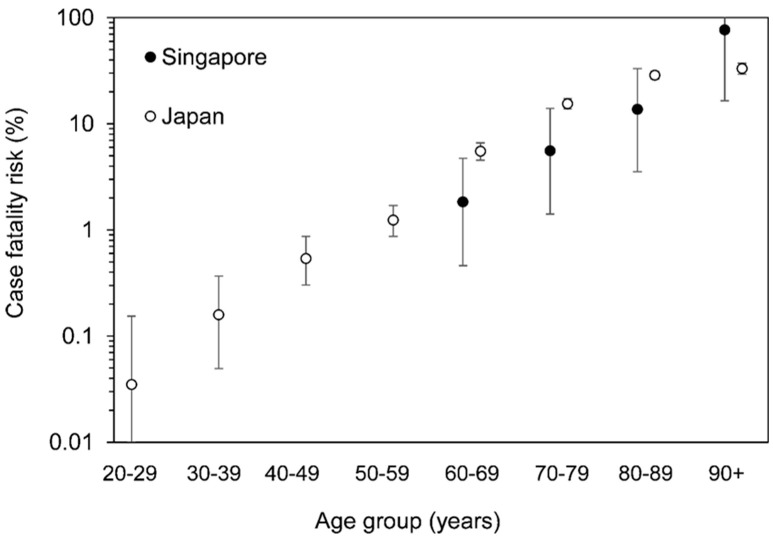
Comparisons between the unbiased case fatality risk of COVID-19 by age group between Singapore and Japan. The unbiased COVID-19 CFR according to age group in Singapore (black dots) and Japan (white dots). Whiskers show the 95% confidence intervals of each CFR.

The age-specific CFRs in Singapore were estimated as 1.84% (95% CI: 0.46–4.72%) among cases aged 60–69 years, 5.57% (1.41–13.97%) among those aged 70–79 years, 13.73% (3.53–33.15%) among those aged 80–89 years, and 76.92% (16.49–100.00%) among those aged 90 years or older. For Japan, the estimated age-specific CFRs were 5.52% (4.55–6.62%) among cases aged 60–69 years, 15.49% (13.81–17.27%) among those aged 70–79 years, 28.77% (26.28–31.35%) among those aged 80–89 years, and 33.22% (29.33–37.27%) among those aged 90 years or older. The two groups are compared visually in Figure 2. The unbiased CFRs among those aged 0–59 years in Singapore and those aged 0–19 years in Japan could not be calculated because of the absence of death in these groups. The SMR of COVID-19 in Japan, when compared to Singapore as the baseline, was estimated at 1.46 (95% CI: 1.09–2.96). The overall unbiased COVID-19 CFR of Japan was higher than that of Singapore.

## 4. Discussion

The present study investigated the disparity in the risk of death from COVID-19 between Singapore and Japan. Although only the 70–79-year age group had 95% CIs that did not overlap between countries, the estimated CFRs for COVID-19 cases in Singapore showed a smaller trend than those in Japan for cases aged 60–89 years. The calculated SMR for COVID-19 indicates that the overall COVID-19 CFR for Singapore is lower than that for Japan. 

One possible explanation for the lower COVID-19 CFR for Singapore compared with Japan is that the COVID-19 cases in Singapore may have been caused by a variant of severe acute respiratory syndrome-coronavirus-2 (SARS-CoV-2), the causative agent of COVID-19 that led to a milder clinical course of infection compared with other SARS-CoV-2 strains [9]. The COVID-19 SMR calculated for Japan in comparison to Singapore reveals a broad difference in the risk of death between these two countries, which supports the possibility of differences in viral strains between these countries. If a low-virulence strain of SARS-CoV-2 is responsible for the difference in COVID-19 CFRs between Japan and Singapore, then successful immunization against COVID-19 may be possible in the future.

Another plausible explanation for the COVID-19 CFR differences between Japan and Singapore is the differing COVID-19 case detection capacities of these countries. Singapore was engaged in extensive contact tracing, partly using digital tracing tools, from a very early stage of the epidemic. In contrast, Japan relied on classic tracing efforts, and there was difficulty in elevating the coverage of mobile phone users who had installed tracing apps [10,11,12]. Thus, it is very likely that some ascertainment bias contributed to the difference in COVID-19 SMR estimated here.

Several limitations of this work must be acknowledged. First, because the dataset for Singapore included cases only through 19 April 2020, our estimates of COVID-19 CFR could have been biased by time. However, as of 2 September 2020, the cumulative number of COVID-19 deaths in Singapore is only 27 persons, so extending the timespan for the dataset of COVID-19 cases in Singapore would have little effect. Second, an external assumption for the delay from illness onset to death was required for the COVID-19 cases in Singapore to address the right censoring during the CFR estimation. The line list of confirmed COVID-19 cases from Singapore rarely contained cases whose dates of illness onset and death were both recorded.

Despite these limitations, the present study clearly demonstrates that the confirmed CFR of COVID-19 for Singapore is lower than that for Japan. Although various underlying explanations can be suggested, the small CFR estimate for Singapore is consistent with the existence of a low-virulence SARS-CoV-2 variant and a high ascertainment rate owing to contact tracing. In the future, the infection fatality risk for COVID-19, with the denominator defined by the number of infections, should be estimated via seroepidemiological investigation [13]. A serosurvey could play an important role in helping to elucidate the entire picture of COVID-19 infection dynamics without ascertainment bias [14,15]. If a low-virulence SARS-CoV-2 strain does still exist, then successful immunization against COVID-19 may be possible.
